# Complicated diverticulitis with colovesical fistula and bladder abscess formation in pregnancy: a case report

**DOI:** 10.1515/crpm-2023-0030

**Published:** 2024-09-04

**Authors:** Shirley Huang, Jiahua Chen, Natalie Rivera, Kavitha T. Ram, Howard L. Minkoff

**Affiliations:** State University of New York (SUNY), Downstate Health Sciences University (Student), Brooklyn, NY, USA; Maimonides Medical Center (The Department of Obstetrics and Gynecology), Brooklyn, NY, USA; State University of New York (SUNY), Downstate Health Sciences University (Faculty, School of Public Health), Brooklyn, NY, USA

**Keywords:** diverticulitis, pregnancy, abdominal pain, abscess, fistula

## Abstract

**Objectives:**

Diverticulitis, characterized by inflammation or infection of diverticula, is rarely observed during pregnancy due to its association with elderly patients. Limited literature exists regarding its diagnosis and management in pregnant patients, especially in the setting of complications.

**Case presentation:**

This paper presents a case of a 37-year-old multiparous woman diagnosed with complicated diverticulitis, including colovesical fistula and bladder abscess formation.

**Conclusions:**

This paper highlights the importance of considering diverticulitis in pregnant patients with abdominal pain, the need for timely diagnosis, and the significance of multidisciplinary care.

## Introduction

Diverticulitis, characterized by inflammation and micro-perforations of colon diverticula, is linked to advanced age and is rarely reported during pregnancy [1]. Diverticulitis clinically manifests with intermittent to constant pain in the lower abdomen and may or may not present with fever, nausea, vomiting, and peritoneal signs [1]. Its prevalence increases with age and therefore, it is more commonly seen in elderly patients over the age of 60 with approximately 5 % of cases occur in adults under the age of 40 [2]. Given the age of pregnant people, this is a disease that is rarely seen within this patient population. Because of the rarity of diverticulitis, and the abundance of etiologies for abdominal pain, the diagnosis of diverticulitis in pregnancy is sometimes delayed or missed. This paper presents the novel case of diverticulitis complicated by a colovesical fistula and bladder wall abscess in a pregnant patient and its management from a multidisciplinary team. This paper also discusses the rarity of diverticulitis in pregnant patients, its increase in incidence in this patient population due to the rise of advanced maternal age, its clinical manifestations, and the challenges of diagnosis and management due to more common alternate possible causes of abdominal pain during pregnancy (Table 1).

**Table 1:** Overview of gestational age, symptoms, and management of the patients presented in the individual case reports 3], [4], [5], [6], [7], [8], [9.

Case report	Gestational age, weeks	Presenting symptoms	Management
Ragu, N., et al.	29	Fever, abdominal pain	Antibiotics
Pelosi, MA., et al.	20	RUQ pain, nausea	Antibiotics, laparoscopy
Sherer, DM., et al.	33	Nausea, vomiting, LLQ pain	Analgesics, laparotomy and Hartmann’s procedure postpartum
Kasaven, LS., et al.	24	Fever, left iliac fossa pain	Antibiotics, ileostomy postpartum
Milczarek-Łukowiak, M., et al.	34	Peritoneal symptoms	Exploratory laparotomy, drain placement
Gendernalik, J., et al.	28	LLQ pain, nausea, vomiting	Fluids, IV pain medication, antibiotics
Bodner, J., et al.	37	Abdominal pain, nausea, vomiting	Antibiotics, hemicolectomy

### Case presentation

**Figure 1:**
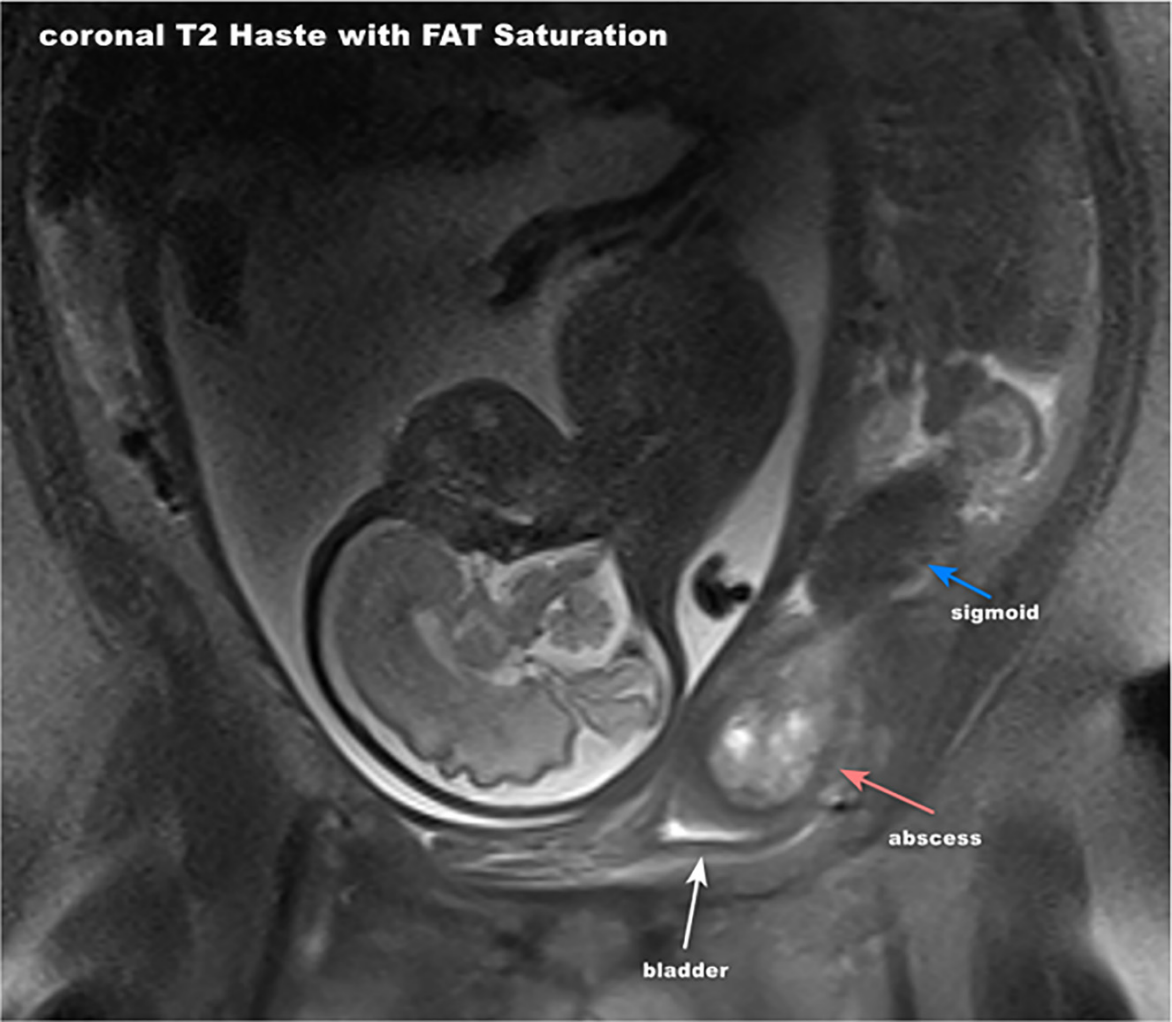
A 6 × 3 × 4.5 cm edematous cystic teardrop shaped structure abutting the sigmoid bowel with surrounding edema visualized on MRI abdomen/pelvis.

A 37-year-old G5P4004 presented to our hospital at 30 w4d with a 3-week history of persistent left lower quadrant pain, which acutely worsened on the day of admission. She denied contractions, vaginal bleeding, or leakage of fluid, and endorsed fetal movement. She also denied fever, chills, nausea, vomiting, and constipation. She endorsed a history of “bowel inflammation” a year ago that was managed with antibiotics. Her obstetrical history was significant for GDMA1, gestational diabetes controlled with diet and exercise, in the current pregnancy, and severe preeclampsia in her second and fourth pregnancies. On physical exam, her abdomen was tender to palpation in the left lower quadrant without rebound. Her pelvic exam revealed a 1 cm long cervix that was unchanged upon reexamination. MRI abdomen/pelvis without IV contrast showed a 6 × 3 × 4.5 cm edematous cystic teardrop shaped structure abutting the sigmoid bowel with surrounding edema to the left of the uterus, which was concerning for ovarian torsion or a torsed pedunculated myoma (Figure 1). The patient was taken to the operating room for a diagnostic laparoscopy. Special measures were taken to ensure the safety of the patient and fetus. The patient was placed on a 15-degree lateral tilt to ensure left uterine displacement for maternal hemodynamics and adequate uteroplacental perfusion. The patient was also placed on a fetal monitor throughout the procedure. NICU was also notified and ready to intervene in case of preterm delivery. Upon entry into the peritoneum, frank purulent discharge was visualized throughout the cavity along with inflammatory changes of the bowel, and a myxoid appearing structure adjacent to the sigmoid colon. General surgery (GS) was consulted intra-operatively. There was no clear evidence of hollow viscous perforation. However, given the patient’s gravid uterus, the bowel could not be run in its entirety. Following the procedure, the patient was started on linezolid and piperacillin-tazobactam as per the infectious disease consultant’s recommendations. A post operative CT abdomen pelvis with PO contrast was obtained per GS’s recommendation. The study was performed using radiation dose reduction software to reduce patient exposure. The scan revealed sigmoid diverticulitis, colonic wall thickening, and pericolonic inflammation. A 5.4 cm × 3.8 cm thick-walled collection, suspicious for abscess, was visualized adjacent to the sigmoid colon. Interventional radiology (IR) was consulted for tentative abscess drainage, which was initially deferred by the team given the overall clinical picture. The patient reported increased pain in her left lower quadrant with up trending white blood count in the days following her surgery, raising suspicion for worsening intra-abdominal infection. A repeat MRI abdomen/pelvis without contrast was ordered on post-operative day 5 showing a thick-walled sigmoid colon with surrounding inflammation, and a colovesical fistula extending from sigmoid colon to the left superior bladder wall with an associated 4.1 × 2.6 × 4.9 cm abscess within the bladder wall, consistent with sequelae of perforated diverticulitis. With the new concerning MRI findings, the patient underwent a drain placement under sonogram guidance by IR near the bladder wall. Drain cultures were sent which revealed the presence of E.coli and Klebsiella Pneumoniae. Drain output was highly suggestive of gastrointestinal content based on its color and the cultured organisms. The patient’s antibiotics were switched to ertapenem for adequate coverage. Urology was also consulted due to the location of the abscess within the bladder wall. The patient remained under the supervision of the Obstetrics team and received multidisciplinary care. She reported significant clinical improvement with a repeat MRI one-week post-drain placement showing interval decrease of the abdominal collection and shrinkage in size of the bladder abscess. Urine cultures were found to be negative. The patient was discharged on hospital day 17 with a plan to keep her drain bag in place until after delivery, and to continue IV ertapenem through a PICC line.

**Figure 2:**
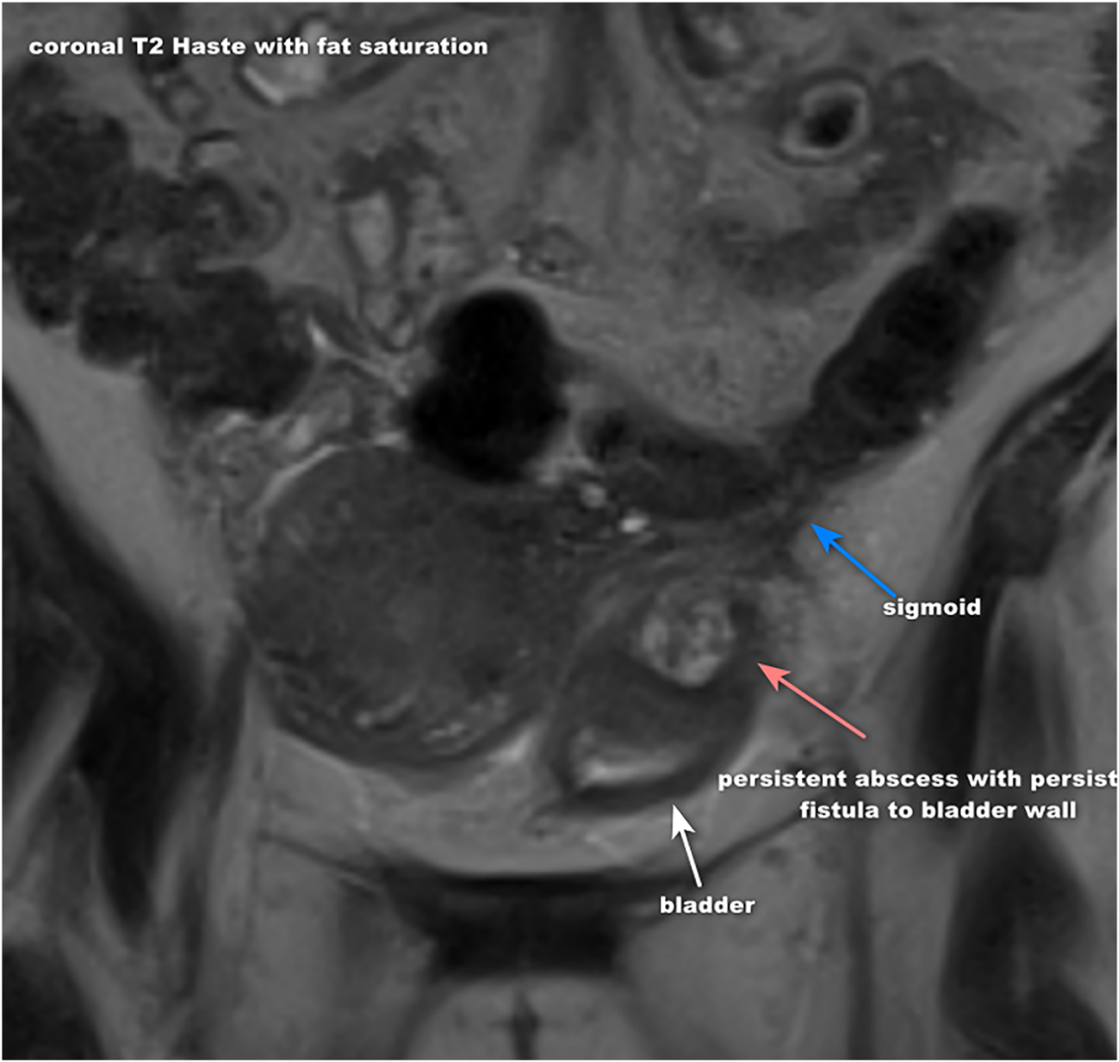
Repeat MRI/abdomen postpartum showing persistent abscess and fistula to the bladder wall.

Approximately 3 weeks following her discharge, the patient returned to the hospital at 35 weeks and 6 days gestational age due to contraction pain and vaginal bleeding. She was found to be 3 cm dilated and 90 % effaced, with the fetal head at −3 station. The patient was admitted for expectant management of labor, and colorectal surgery was alerted for possible operative intervention should she require a c-section. The patient had a vaginal delivery of a vigorous female neonate with Apgar scores of 9 and 9 at 1 and 5 min. Her postpartum course was complicated by retained placenta which required evacuation of the uterine cavity via manual vacuum aspiration in the operating room. An immediate postpartum CT scan showed significant decrease in intra-abdominal collection. The patient continued her daily IV antibiotics and remained clinically stable. She was discharged on hospital day 2 with follow up with colorectal surgery and infectious disease. Repeat MRI of the abdomen/pelvis following discharge showed persistence of the abscess and fistula to the bladder wall (Figure 2). The patient did not complete her postpartum visit however, continued to follow up with colorectal surgery and underwent a robotic assisted sigmoid colectomy with end-to-end anastomosis and ureteral stent placement bilaterally.

## Discussion

The PubMed database was used to search for case reports of pregnant patients with diverticulitis. Search words included, but were not limited to, diverticulitis, diverticulitis in pregnancy, complicated diverticulitis in pregnancy, and diverticulitis and fistula formation. Given the number of existing case reports found, articles from all years were considered. Journal articles were included if they were accessible through PubMed or the institutional library. Additional articles and references were found through the reference section of identified articles. While Meckel’s diverticulitis in pregnancy is widely discussed in the literature, there are few case reports describing diverticulitis of the colon in pregnant woman. Our research yielded seven case reports 3], [4], [5], [6], [7], [8], [9 all of which were included in a literature review [2] summarizing a total of twelve cases. The patients described in the seven case reports ranged in age from 33 to 38 3], [4], [5], [6], [7], [8], [9. They presented with symptoms including, but not limited to, fever, chills, nausea, vomiting, and abdominal pain 3], [4], [5], [6, 8], [9. Symptoms varied between patients with abdominal pain being the most reported symptom 3], [4], [5], [6, 8], [9, similar to our patient who initially presented with a history of left lower quadrant pain that had acutely worsened. Further work up of the patients described in the case reports proved essential as some of the initial differentials included cholecystitis or appendicitis [5, 9]. Patients were diagnosed with various imaging modalities such as ultrasound, MRI, and CT as well as surgical procedures including laparotomy 3], [4], [5], [6], [7], [8], [9. After diagnosis, patients were either treated medically with an antibiotic regimen, surgically, or a combination of both. Further complications described in these case reports included abscess formations [3, 9], adhesion formation [4], and septicemia [6]. In our patient, we saw a combination of these complications with the formation of a persistent colovesical fistula and a bladder wall abscess, unique to previously described cases in literature. This emphasizes the importance of receiving care from a multidisciplinary team as any delays or failure of proper management could produce further consequences including, but not limited to, chronic urinary tract infections, pyelonephritis, as well as adhesion and stricture formation. Throughout this patient’s hospital course, she was seen, evaluated, and followed by multiple teams including obstetrics, general surgery, interventional radiology, and urology. Furthermore, while this patient received treatment comprised of laparoscopic surgery, an antibiotics regimen, and drain placement, given the limited literature that exists, it is difficult to assess the efficacy and outcomes of her treatment plan versus that of others, especially in the setting of multiple complications.

Besides maternal outcomes, given the paucity of literature, it is challenging to evaluate fetal outcomes as well. As per the case reports, the modes of delivery included both vaginal and cesarean section and the gestational age of the fetus ranged from 20 to 37 weeks 3], [4], [5], [6], [7], [8], [9. However, it is important to note that there is little to no mention of birth planning amongst these patients in these case reports or follow up regarding long-term complications of these neonates.

While diverticulitis in pregnancy is seemly rare, the exact incidence is currently unknown. However, this number may only rise with time as the incidence of diverticulitis in younger patients continues to rise over the years. Also, given that the increase in the prevalence of diverticulitis with age, and the rise in advanced maternal age, as reflected by increased birth rates for women aged 35–44 this past decade, it stands to reason that there will be an increase in the incidence of diverticulitis in pregnant woman [10].

It is important to take this information into consideration given the lack of a defining presentation of diverticulitis. As described in the literature and seen in our own patient, the most reported symptom is abdominal pain. The sole complaint of acute abdominal pain in pregnancy can lead to a very wide differential including, but not limited to appendicitis, cholecystitis, round ligament pain, and constipation. These diagnoses typically fall higher on the list of differentials as such conditions are more commonly seen within this age group and patient population. A complete clinical picture including the history, review of systems, physical exam, and lab work should be used to narrow down the differential as diverticulitis may also present with fever, nausea, vomiting, an elevated white count, and intraperitoneal free air. Even with a thorough evaluation, the diagnosis may still be inconclusive.

As seen in this patient, and others in the reported cases, diverticulitis in pregnancy is a challenging disease to both diagnose and manage. Improved management will require greater understanding of the prevalence, clinical course, and full impact of diverticulitis within this patient population.

## Conclusions

Given the rapid progression and serious sequelae of diverticulitis, as described in literature as well as seen in this patient’s hospital course, timely consideration of diverticulitis in pregnant patients presenting with abdominal pain is essential. With increasing maternal age, there could be a higher occurrence of diverticulitis during pregnancy. The complexities of diagnosing and managing diverticulitis especially in the setting of complications in this population underscore the need for multidisciplinary care to ensure maternal and fetal well-being.
